# An Integrated Physical Therapy Using Spencer’s Technique in the Rehabilitation of a Patient With a Frozen Shoulder: A Case Report

**DOI:** 10.7759/cureus.41233

**Published:** 2023-06-30

**Authors:** Pratik Phansopkar, Moh'd Irshad Qureshi

**Affiliations:** 1 Musculoskeletal Physiotherapy, Ravi Nair Physiotherapy College, Datta Meghe Institute of Higher Education and Research (DU), Wardha, IND; 2 Neuro-Physiotherapy, Ravi Nair Physiotherapy College, Datta Meghe Institute of Higher Education and Research (DU), Wardha, IND

**Keywords:** spadi, manual therapy, rehabilitation, spencer technique, frozen shoulder

## Abstract

A frozen shoulder is a painful condition characterized by pain and stiffness. In frozen shoulder, the capsule of the joint gets inflamed, leading to pain that limits shoulder movement and thereby affects shoulder functions. There are three stages of frozen shoulder, and the symptoms differ according to the stage. Physiotherapy plays an important role in the management of a frozen shoulder. The Spencer technique is a seven-step technique that is used to treat shoulder movement restrictions. In this case report, we present the case of a 57-year-old male shopkeeper by occupation with a right frozen shoulder with complaints of pain and stiffness around the shoulder region and reduced range of motion (ROM) for more than six weeks. Physiotherapy rehabilitation was given to the patient using Spencer’s technique along with standard management for three weeks. Significant improvement in range of motion, decrease in pain, and functional disability, i.e., shoulder pain and disability index (SPADI), were seen post-rehabilitation and were sustained when evaluated at the end of the second, third, and sixth months.

## Introduction

A frozen shoulder is characterized by pain and stiffness in the Glenohumeral (GH) joint, which is characterized by joint hypomobility [1]. In 10% to 40% of frozen shoulder cases, bilateral involvement of the shoulder may occur [1]. The frozen shoulder was first identified as periarthritis affecting the shoulder's periarticular soft tissues [2]. Periarthritis consists of sub-synovial tissue that is characterized by a non-specific chronic inflammatory reaction that triggers the capsular and synovial thickening associated with it. Injections of corticosteroids have long been used to treat frozen shoulder joints, adjacent soft tissues, or the subacromial bursa, which was traditional management to address only pain [2]. However, with the evolution of physical therapy research, the major emphasis is on physiotherapy approaches to treating frozen shoulders rather than painkillers or injections. Mobilization techniques applied early at the onset of the frozen shoulder have been extensively studied, and the emphasis on involving gentle range of motion (ROM) exercises in physiotherapy management has shown their vital role in bringing about successful recovery in frozen shoulder patients [2].

The GH joint is impaired by the frozen shoulder and restricts active and passive motion due to adhesion and fibrosis in the GH capsule, which reduce joint space. Three phases of frozen shoulder include stage 1 freezing, stage 2 frozen, and stage 3 resolution of the process, i.e., thawing. Risk factors such as extended immobilization, relatively minor trauma, and surgical trauma have been associated with the frozen shoulder. Adhesive capsulitis is associated with medical conditions such as diabetes, hyperthyroidism, coronary artery disease, inflammatory arthritis, and cervical spondylosis [3].

Physiotherapy is an important part of the healthcare system and consists of various techniques to rehabilitate patients using electrotherapy, exercise therapy, and manual therapy. In the frozen shoulder, the mobility of the joint is affected, and physiotherapy helps in improving the range by various methods, namely Maitland, Kaltenborn, and Mulligan's mobilizations [3]. Spencer’s technique is a mobilization technique that consists of seven-step techniques that are used to improve mobility and reduce the stiffness of the joint [4]. The patient with a frozen shoulder mainly complains of stiffness and pain in the shoulder joint. In this case report, we present a frozen shoulder patient managed with Spencer's technique and not only evaluated post-rehabilitation effects but also evaluated its sustainability at various timelines by monitoring the shoulder's active range of motion, visual analogue scale (VAS) for pain, and shoulder functionality by shoulder pain and disability index (SPADI).

## Case presentation

A 57-year-old male came to physiotherapy OPD with the complaint of right shoulder pain (anterolateral aspect) and difficulty in shoulder movements for more than six weeks. The patient was in a high sitting posture during the examination. Physical examination revealed normal vital signs, including no temperature variations, a pulse rate of 68 beats/minute, a respiratory rate of 24 breaths/minute, and a blood pressure of 110/70 mmHg. Grade 2 tenderness was present at the right shoulder joint on the anterolateral aspect. The patient was unable to walk with a normal arm swing and was unable to ride his two-wheeler appropriately, which suggests that the shoulder discomfort was affecting his daily activities. Pain and stiffness were present with passive and active movement (Table 1). Active shoulder range of motion, pain on rest, and activity were assessed using a VAS; functional disability was evaluated with the SPADI at various timelines (Table 2).

**Table 1:** Pre-treatment shoulder range of motion assessment

Movements	Left	Right (affected)
Flexion	0–170˚	0–117˚
Extension	0–45˚	0–22˚
Medial rotation	0–65˚	0–18˚
Lateral rotation	0–80˚	0–33˚
Abduction	0–150˚	0–90˚
Adduction	0–30˚	0–20˚

**Table 2:** Post-treatment outcome measures

Outcomes for right shoulder	Day 1	Day 21	2^nd^ month	3^rd^ month	6^th^ month
Range of motion					
Flexion	0–117˚	0–155˚	0–157˚	0–158˚	0–158˚
Extension	0–22˚	0–32˚	0–34˚	0–34˚	0–34˚
Medial rotation	0–18˚	0–35˚	0–37˚	0–38˚	0–38˚
Lateral rotation	0–33˚	0–50˚	0–52˚	0–52˚	0–52˚
Abduction	0–90˚	0–134˚	0–136˚	0–137˚	0–137˚
Adduction	0–20˚	0–25˚	0–26˚	0–26˚	0–26˚
Pain on					
Rest	7/10	3/10	2/10	1/10	0/10
Activity	8/10	4/10	3/10	3/10	2/10
SPADI	72%	35%	34%	32%	30%

Treatment

**Figure 1:**
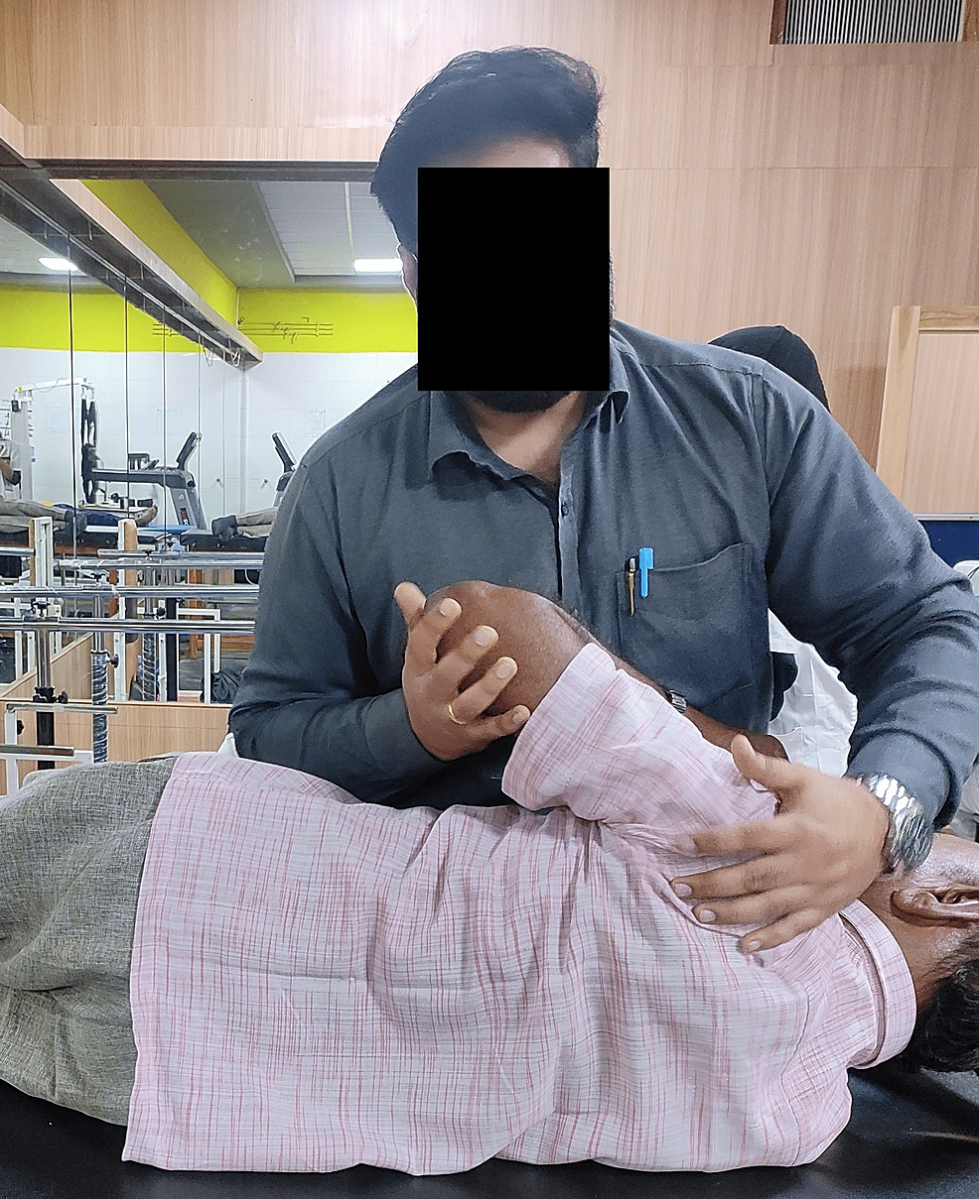
Spencer technique for phase 1 frozen shoulder

**Figure 2:**
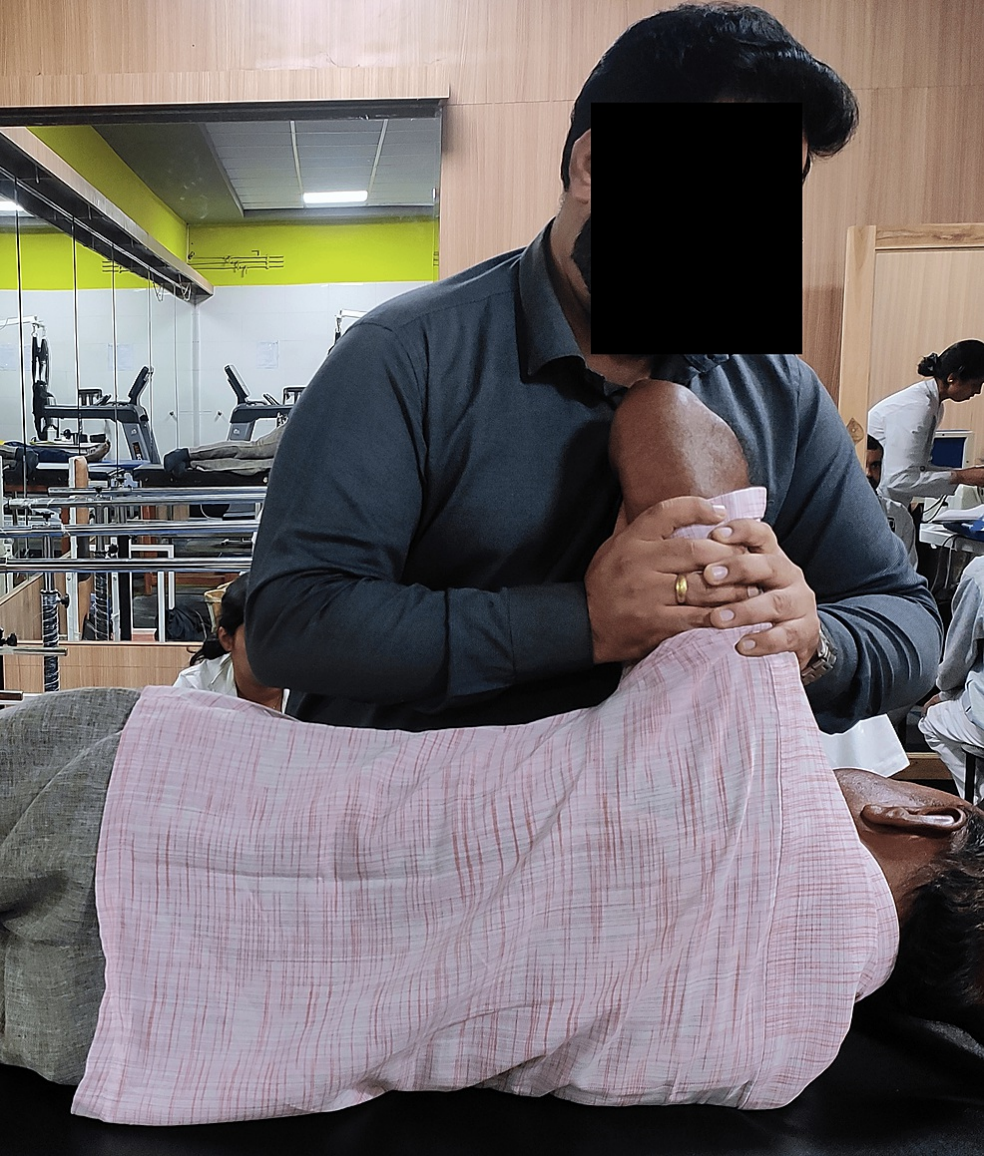
Spencer technique for phase 2 frozen Shoulder

According to current studies, the pathophysiology and neuroscience underlying the disease are the most successful ways to alleviate catastrophic pain symptoms in patients with frozen shoulders [2]. The primary goal of rehabilitation in the initial phase is to maintain the available range and decrease the pain. Scapular-setting exercises are given to avoid wasting time. In the second phase, Spencer’s technique of mobilization was given to improve the range of motion along with interferential therapy (IFT) (Figures 1-2). In the third phase, Spencer’s technique was continued to further improve the range and reduce stiffness (Table 3).

**Table 3:** Week-wise physiotherapy treatment given to patient

Timeline	Intervention	Dosage
0-1 weeks	Moist heat therapy has given using a hot pack	20 minutes
	Codman’s exercises were given in which the patient was asked to stand and flex the trunk from the waist with the support of an uninvolved hand and the affected arm hanging downwards, the patient was asked to rock the body in a circular pattern without contracting shoulder muscles. The arm was moved from side to side, back and forth, and in the clockwise and anti-clockwise directions.	1 set 5 sessions/week [3]
	Scapular setting exercises were given namely - shoulder shrugs were given where the patient was asked to lift shoulders straight and hold in this position for 5 sec and then back to a neutral position. Shoulder retraction was given where the patient was asked to pull the shoulder blades in towards each other hold this for 5 sec and then back to neutral.	1 set 5 sessions/week [3]
	Interferential therapy was applied around the right shoulder in a clover leaf pattern where the electrode of each pair was placed diagonally opposite (superior-inferior, anterior-posterior) to one another with a beat frequency of 100 Hz.	15 minutes [3]
	Spencer’s technique was performed in which the patient’s elbow was flexed and 90˚ abduction of the shoulder was done. The humerus was rotated both clockwise and anticlockwise by using the elbow as a pivot. In the next step, the patient's elbow was flexed and the shoulder was kept in an abducted position and the traction force was exerted on the shoulder joint while rotating the humerus clockwise and anti-clockwise.	2 sets of 10 repetitions with a rest of 1 minute between sets for 5 sessions/week [4]
2-3weeks	Moist heat therapy	20 minutes
	Codman's exercise progression was done by increasing the repetitions.	2 set 5 sessions/week
	Spencer technique progression was done with an increase in the number of sets.	3 sets for 10 repetitions with 1-minute rest between sets for 5 sessions/week
	Interferential therapy	15 minutes

## Discussion

In this case report, we have utilized the Spencer technique for a frozen shoulder patient, and its effect was evaluated on pain and stiffness in the frozen shoulder. We also evaluated its efficacy and whether the effects achieved were sustained or not, which is a major concern in terms of relapse of stiffness in this kind of patient. Frozen shoulder is treated mainly with analgesics and physiotherapy with shoulder mobilization exercises [5]. A periarticular hydrocortisone injection is sometimes given to relieve pain and inflammation. Physiotherapy plays an important role in the prevention and resolution of this condition. Regular practise of particular movements could be instrumental in the prevention of primary capsulitis, whereas in secondary capsulitis, careful early mobilization to the extreme ROM needs to be emphasized for the other benefits of exercise in addition to the prevention of secondary adhesive capsulitis [6]. Our primary aim is to reduce pain, increase the extensibility of the shoulder capsule, improve the strength of the muscles, and improve the mobility of the shoulder. Superficial heating modalities such as hot packs, IFT, ultrasound therapy, and gentle, relaxed passive movements are used to reduce pain and increase the extensibility of the contracted soft tissues. The strength of the muscles can be increased by resistance exercises and isometrics [7].

To create a low-frequency effect, interferential therapy works by passing two medium-frequency alternating currents across one another. Interferential currents help with decreasing pain, activating muscles, increasing blood flow, and reducing edoema, which has had a positive outcome in our patient in reducing shoulder pain and muscle spasm [8,9].

Pendular exercises, shoulder pulleys, shoulder wheels, finger ladders, wall wash exercise stretching, and mobilization are used to increase joint mobility.

The main extraarticular factors that limit motion may include adhesions and collagen cross-linkages in the ligament and joint capsule. These cross-linkages of collagen, which form due to a decrease in glycosaminoglycans (GAG), could be broken by joint mobilization to improve excursion [8]. Joint mobilization may certainly affect both the synovial fluid and the joint capsule [10].

In Spencer's technique, there are seven steps as follows: shoulder extension along with elbow flexion, shoulder flexion with elbow extension, circumduction with compression, circumduction with distraction, shoulder abduction and internal rotation with elbow flexion, shoulder adduction and external rotation with elbow flexion, stretching tissue and pumping fluids with the arm extended [11].

Spencer's method improves pain-free movement by stretching the capsule of the shoulder and constricted soft tissues, regaining specific joint mobility while reducing pain by modifying the circulatory pain biomarkers. This method enhances lymphatic flow away from the area of treatment [12]. This technique restores the joint's normal range of motion and resets neural reflexes. The joint structure's nutrition, such as the capsule, glenoid labrum, articular surfaces, circulation, and lubrication, is improved by passive, repetitive translation movements, traction, or gliding. Arthokinematic glide and roll motion are regained, and the pathomechanical alterations in the joint are repaired. Increased accessory movement, such as gliding, promotes the recovery of shoulder mobility by allowing the osteokinematic glenohumeral rotation to return to normal biomechanics [11].

A reduction in pain after the mobilization of the joint occurs due to various mechanisms, such as neurophysiological effects occurring by the activation of type II mechanoreceptors and by the hindrance of type IV nociceptors [13], Golgi tendon organ activation, and reflex inhibition of the muscle at the end of the passive joint mobilization [14]. Joint mobilization lowers muscle activity, reducing periarticular tissue discomfort, muscular tension, and muscle concentric activation [15].

Khyathi et al. conducted a single-blind study to find the effects of the Spencer technique and Mulligan's technique on patients with frozen shoulders and reported that both treatment approaches are effective in having short-term effects on reducing pain, improving shoulder mobility, and functional disability; however, this study was not aimed at evaluating long-term effects [4]. Simrat et al. utilized the Spencer technique on a chronic shoulder injury related to vaccine administration and reported the technique being beneficial in improving shoulder function; however, the improvements were not monitored for the long term to evaluate its sustainability, and they emphasised the need to study this novel technique in the future, which would prove beneficial in frozen shoulder management [16].

## Conclusions

This case report utilized a novel Spencer technique to address a frozen shoulder patient. The Spencer technique utilized has a significant effect on improving the shoulder range of motion, decreasing pain, and further reducing the functional disability associated with a frozen shoulder, and the improvements were sustained post-treatment when evaluated at various timelines that are the second, third, and sixth months. Physiotherapy plays a vital role in treating frozen shoulders, and involving this technique can be beneficial to not only having immediate effects but also sustaining them for the long term. Further studies should emphasize comparing this technique with the traditionally used methods of mobilizing the shoulder joint in frozen shoulders.

## References

[1] Chan HB, Pua PY, How CH (2017). Physical therapy in the management of frozen shoulder. Singapore Med J.

[2] Dias R, Cutts S, Massoud S (2005). Frozen shoulder. BMJ.

[3] Jason JI, Sundaram SG, Subramani MV (2015). Physiotherapy interventions for adhesive capsulitis of shoulder: a systematic review. Int J Physiother Res.

[4] Khyathi P, Babu VK, Kumar SN, Asha D (2015). Comparative effect of Spencer technique versus Mulligans technique for subjects with frozen shoulder a single blind study. Int J Physiother.

[5] Duzgun I, Turgut E, Eraslan L, Elbasan B, Oskay D, Atay OA (2019). Which method for frozen shoulder mobilization: manual posterior capsule stretching or scapular mobilization?. J Musculoskelet Neuronal Interact.

[6] Almureef SS, Ali WM, Shamsi S, Bakheet M (2020). Effectiveness of mobilization with conventional physiotherapy in frozen shoulder: a systematic review. Int J Recent Innov Med Clin Res.

[7] Patil A, Jawade S, Chitale N (2021). Physiotherapy rehabilitation in arthroscopic rotator cuff repair patient: a case report. J Pharmaceutical Research Int.

[8] Gaba E, Sethi J, Bhardwaj M (2020). Effect of interferential therapy over ultrasound therapy with common protocol of manual therapy in Grade - II frozen shoulder. J Exerc Sci Physiother.

[9] Madhuripu P, Kumar MP (2022). Effect of IFT with anterior glide versus posterior glide joint mobilisation technique on shoulder external rotation ROM in patients with adhesive capsulitis: comparative study. Indian J Physiother Occup Ther Print.

[10] Grubbs N (1993). Frozen shoulder syndrome: a review of literature. J Orthop Sports Phys Ther.

[11] Haveela B, Dowle P, Chandrasekhar P (2018). Effectiveness of Mulligan’s technique and Spencer’s technique in adjunct to conventional therapy in frozen shoulder: a randomised controlled trial. Int J Adv Res Dev.

[12] Jivani RR, Hingarajia DN (2021). Effect of Spencer muscle energy technique versus Maitland’s mobilization technique on pain, ROM and disability in patients with frozen shoulder: a comparative study. Int J Physiother Res.

[13] Mangus B, Hoffman L, Hoffman M, Altenburger P (2023). Basic principles of extremity joint mobilization using a Kaltenborn approach. Int J Sports Rehabil.

[14] Lundberg A, Malmgren K, Schomburg ED (1978). Role of joint afferents in motor control exemplified by effects on reflex pathways from Ib afferents. J Physiol.

[15] Zusman M (1986). Spinal manipulative therapy: review of some proposed mechanisms, and a new hypothesis. Aust J Physiother.

[16] Veera S, Chin J, Kleyn L, Spinelli S, Tafler L (2020). Use of osteopathic manipulation for treatment of chronic shoulder injury related to vaccine administration. Cureus.

